# Halocarbon emissions by selected tropical seaweeds: species-specific and compound-specific responses under changing pH

**DOI:** 10.7717/peerj.2918

**Published:** 2017-01-25

**Authors:** Paramjeet Kaur Mithoo-Singh, Fiona S.-L. Keng, Siew-Moi Phang, Emma C. Leedham Elvidge, William T. Sturges, Gill Malin, Noorsaadah Abd Rahman

**Affiliations:** 1 Institute of Ocean and Earth Sciences (IOES), University of Malaya, Kuala Lumpur, Malaysia; 2 Institute of Biological Sciences, Faculty of Science, University of Malaya, Kuala Lumpur, Malaysia; 3 Institute of Graduate Studies (IGS), University of Malaya, Kuala Lumpur, Malaysia; 4 Centre for Ocean and Atmospheric Sciences, School of Environmental Sciences, University of East Anglia, Norwich Research Park, Norwich, United Kingdom; 5 Department of Chemistry, University of Malaya, Kuala Lumpur, Malaysia

**Keywords:** Halocarbons, Emission rate, Seawater pH, Tropical seaweeds, Climate change, *Kappaphycus*

## Abstract

Five tropical seaweeds, *Kappaphycus alvarezii* (Doty) Doty ex P.C. Silva, *Padina australis* Hauck, *Sargassum binderi* Sonder ex J. Agardh (syn. *S. aquifolium* (Turner) C. Agardh), *Sargassum siliquosum* J. Agardh and *Turbinaria conoides* (J. Agardh) Kützing, were incubated in seawater of pH 8.0, 7.8 (ambient), 7.6, 7.4 and 7.2, to study the effects of changing seawater pH on halocarbon emissions. Eight halocarbon species known to be emitted by seaweeds were investigated: bromoform (CHBr_3_), dibro­momethane (CH_2_Br_2_), iodomethane (CH_3_I), diiodomethane (CH_2_I_2_), bromoiodomethane (CH_2_BrI), bromochlorometh­ane (CH_2_BrCl), bromodichloromethane (CHBrCl_2_), and dibro­mochloromethane (CHBr_2_Cl). These very short-lived halocarbon gases are believed to contribute to stratospheric halogen concentrations if released in the tropics. It was observed that the seaweeds emit all eight halocarbons assayed, with the exception of *K. alvarezii* and *S. binderi* for CH_2_I_2_ and CH_3_I respectively, which were not measurable at the achievable limit of detection. The effect of pH on halocarbon emission by the seaweeds was shown to be species-specific and compound specific. The highest percentage changes in emissions for the halocarbons of interest were observed at the lower pH levels of 7.2 and 7.4 especially in *Padina australis* and *Sargassum* spp., showing that lower seawater pH causes elevated emissions of some halocarbon compounds. In general the seaweed least affected by pH change in terms of types of halocarbon emission, was *P. australis*. The commercially farmed seaweed *K. alvarezii* was very sensitive to pH change as shown by the high increases in most of the compounds in all pH levels relative to ambient. In terms of percentage decrease in maximum quantum yield of photosynthesis (*F*_v_∕*F*_m_) prior to and after incubation, there were no significant correlations with the various pH levels tested for all seaweeds. The correlation between percentage decrease in the maximum quantum yield of photosynthesis (*F*_v_∕*F*_m_) and halocarbon emission rates, was significant only for CH_2_BrCl emission by *P. australis* (*r* = 0.47; *p* ≤ 0.04), implying that photosynthesis may not be closely linked to halocarbon emissions by the seaweeds studied. Bromine was the largest contributor to the total mass of halogen emitted for all the seaweeds at all pH. The highest total amount of bromine emitted by *K. alvarezii* (an average of 98% of total mass of halogens) and the increase in the total amount of chlorine with decreasing seawater pH fuels concern for the expanding seaweed farming activities in the ASEAN region.

## Introduction

Marine algae are an important source of biogenic halocarbons, contributing to an approximate 70% of the world’s bromoform (Carpenter & Liss, 2000). The emissions of these volatile halocarbons create a pool of atmospheric halogen radicals, which directly or indirectly contribute to climate change. The vast majority of halocarbons emitted by marine algae are relatively short-lived in the atmosphere (Leedham et al., 2013). Whereas the emission of such short-lived gases at mid and high latitudes is of little consequence to stratospheric chemistry, emissions in the tropics may be transported by tropical deep convection sufficiently quickly to the tropical lower stratosphere to have a significant impact on global stratospheric ozone (Carpenter & Reimann, 2014). Farming of seaweeds in the tropics is expanding rapidly, especially for the cultivation of *Eucheuma* spp. and *K. alvarezii* in the Philippines and Indonesia, which together constitute 33% of global seaweed production. In the last decade, seaweed farming in Indonesia increased 28-fold, with an expected increase in flux of short-lived halogens to the stratosphere (FAOSTAT, 2015; Radulovich et al., 2015). Therefore, we examine the potential effect of seawater pH changes on the release of short-lived halocarbons by five tropical seaweed species from four genera (*Kappaphycus alvarezii*, *Padina australis*, *Sargassum binderi*, *Sargassum siliquosum* and *Turbinaria conoides*). The selected species are both, native and introduced commercially important macrophyte species in tropical South-East Asia.

Algae produce halocarbons when the defense mechanism is activated against mechanical and chemical stress (Ohsawa et al., 2001; Manley, 2002; Paul, De Nys & Steinberg, 2006). Halocarbons emitted by seaweeds have anti-herbivory (McConnell & Fenical, 1977) and antibacterial activities (Neidleman & Geigert, 1986). Brown seaweeds from the temperate region including *Laminariales* and *Fucus*, release large amounts of the iodinated compound, CH_2_I_2_ (Carpenter et al., 2000). We previously reported that brown seaweeds, namely *Sargassum binderi*, *Padina australis*, and *Turbinaria conoides*, which were dominant in a tropical coral reef, emitted various volatile halogenated compounds including tribromomethane (CHBr_3_), dibromomethane (CH_2_Br_2_), iodomethane (CH_3_I), diiodomethane (CH_2_I_2_), bromoiodomethane (CH_2_BrI), bromochloromethane (CH_2_BrCl), bromodichloromethane (CHBrCl_2_), and dibromochloromethane (CHBr_2_Cl) (Keng et al., 2013). Relatively high emissions of CHBr_3_ by the farmed species of *Gracilaria changii* (1,037–1,272 pmol gFW^−1^ h^−1^) and *Kappaphycus alvarezii* (479–558 pmol gFW^−1^ h^−1^) were observed (Leedham et al., 2013); whereby FW refers to the fresh weight of seaweeds measured immediately post incubation.

Decreasing pH had been recorded on the oceans’ surface at a steady rate of 0.0019 each year, caused by an increase in atmospheric CO_2_ concentration (Dore et al., 2009). The coastal marine environment is subjected to constant pH fluctuations, especially in bays, lagoons and tidal pools (Macedo et al., 2001). The effect of pH and CO_2_ concentration on marine algae had been reported (Kuffner et al., 2008; Kroeker et al., 2010; Diaz-Pulido et al., 2011), though not much emphasis has been given to the effect on halocarbon emissions by different seaweed species. Algal growth in terms of allocation of carbon, availability of carbon and essential nutrients is affected by pH changes through its effect on photosynthesis (Juneja, Ceballos & Murthy, 2013). A previous study on the red seaweed, *Eucheuma denticulatum*, found a varying response to pH: CH_2_I_2_ emitted correlates positively with increasing pH (8.2–8.8 using an acid–base titration method) whilst the opposite was observed for CHBr_3_ and CHBr_2_Cl under the same changes (Mtolera et al., 1996). Halocarbon emission by algae is also affected by irradiance (Nightingale, Malin & Liss, 1995; Mtolera et al., 1996; Laturnus et al., 2000; Keng et al., 2013); desiccation (Nightingale, Malin & Liss, 1995; Leedham Elvidge et al., 2015); oxidative stress (Abrahamsson et al., 2003); tissue age (Nightingale, Malin & Liss, 1995); tissue wounding/grazing (Nightingale, Malin & Liss, 1995; Sundström et al., 1996) and photosynthetic activity (Sundström et al., 1996; Goodwin, North & Lidstrom, 1997). The way irradiance affects the emission of halocarbons by seaweeds could be caused by changes in photosynthetic activity (Goodwin, North & Lidstrom, 1997; Ekdahl, Pedersén & Abrahamsson, 1998; Laturnus et al., 2000). This is because halocarbons may be formed to scavenge the hydrogen peroxide produced during the pseudocyclic photophosphorylation or Mehler reaction (Pedersén et al., 1996; Manley & Barbero, 2001). Keng et al. (2013) found that the release of certain brominated and iodinated compounds is positively correlated with the *F*_v_∕*F*_m_ values of brown seaweeds, implying a role of vanadium-containing bromoperoxidases in handling oxidative stress (Winter & Moore, 2009; La Barre et al., 2010).

Rates of halocarbon production may vary based on type of species and geographical locations. There is inadequate research regarding the influence of environmental factors such as pH on halocarbon emissions by seaweeds, especially in the tropics, where rapid transport of volatile compounds to the troposphere occurs due to high sea surface temperatures (Levine et al., 2008). The pH of tropical surface waters has decreased to below 8.1 at present times and is predicted to decrease further to 7.7 by 2100 (Stocker et al., 2013). This paper reports on the effect of changes in seawater pH (following different IPCC scenarios) on the emission of halocarbons by five common tropical seaweeds in Malaysia. The main hypothesis here is that decreases in seawater pH may lead to increases in halocarbon emissions by tropical seaweeds.

## Materials and Methods

### Sample collection and preparation

Five seaweed species were selected for this study, one rhodophyte and four phaeophytes. The rhodophyte *Kappaphycus alvarezii* (Doty) Doty ex P.C. Silva (KLU PSM12876) was purchased from a seaweed farm located near Bum Bum Island, Semporna, Sabah (N04.500°, E118.635°). The *Kappaphycus* species was selected as it is the most commonly cultivated commercial species in the Coral Triangle (Lim et al., 2014). The voucher specimens of the phaeophytes *Padina australis* Hauck (KLU PSM 12877), *Sargassum binderi* Sonder ex J. Agardh (syn.* Sargassum aquifolium* (Turner) C. Agardh) (KLU PSM12878), *Sargassum siliquosum* J. Agardh (KLU PSM12879) and *Turbinaria conoides* J. Agardh Kützing (KLU PSM12880) that were collected from a fringing coral reef at Cape Rachado (N2.417°, E101.853°) and Pantai Purnama (N2.441°, 101.855°) Port Dickson, west coast Peninsular Malaysia, have been deposited in the University of Malaya Seaweeds and Seagrasses Herbarium. These locations are not marine protected areas, allowing access to scientific activities. The phaeophytes were selected based on their abundance and ecological importance in the coral reefs (Wong & Phang, 2004; Keng et al., 2013). Both *S.  binderi* and *T. conoides* were among the top five most frequently occurring (frequency of 37 and 43% respectively) and dominant species (dominance of 33 and 8% respectively) at the sampling sites based on previous 15-month field surveys (Wong & Phang, 2004; Keng, 2013; Keng et al., 2013). All samples were transported back to the laboratory in an ice-chest, cleansed of epiphytes and debris, and cultured in large open aerated tanks with water circulation system. Tanks were filled with unfiltered natural seawater at the University of Malaya hatchery for no longer than three weeks before use.

Before performing the incubation experiments, the seaweeds were gently brushed to remove all epiphytes. Undamaged and complete plants were selected whenever possible for experimental use. In rare cases where plants were cut using razor blade, they were left to recover in seawater under controlled conditions in the hatchery tank system for at least two days to minimize the effect of mechanical stress. All plants were acclimatized under the following conditions: temperature of 30 ± 1 °C, irradiance of 85 ± 5 µmol photons m^−2^s^−1^, 12 h light: 12 h dark cycle, and average salinity of 30 ± 2 PSU; for 16–24 h in an incubator (SSI5R-HS Shel Lab) prior to the experiment.

### HEPES buffer stability test

A four-hour investigation using *S. siliquosum* was done to observe the stability of the zwitterionic HEPES buffer, 4-(2-Hydroxyethyl) piperazine-1-ethanesulfonic acid at the desired pH values. The HEPES buffer is generally non-toxic to organisms including algae (McFadden & Melkonian, 1986; Dias, Barbugli & Vergani, 2016), and was used to prepare the seawater with various pH levels. The two treatments used included seawater (control) and seawater with seaweeds; without any aeration; the pH levels tested were pH 7.2 and 8.0, representing the two extreme pH levels to be used in later experiments that tested pH units of 8.0, 7.8, 7.6, 7.4 and 7.2. All treatments were conducted in triplicates. This pH range was chosen as pH 7.8 was taken as the ambient pH level at the coral reef where the seaweeds inhabit (Hamzah et al., 2011), while the IPCC 2013 report indicates that the pH of tropical surface waters has decreased to below 8.1 at present times and is predicted to decrease further to 7.7 by 2100 (Stocker et al., 2013). Results from the buffer stability test showed that within four hours, there was minimal change in pH (decrease of 0.01–0.05 units) when no aeration was supplied to the flasks containing seaweeds at various pH levels (Table S1). This unaerated condition was then set for the pH incubation studies.

### Study on effect of pH on halocarbon emissions from selected seaweeds

All experiments were conducted in 0.5 L borosilicate flasks (Schott Duran) with glass stoppers and filled with filtered (0.7 µm GF/F, Whatman) natural seawater. Seawater used throughout the experiment was collected from Port Dickson. A set of four sample flasks (filtered seawater with seaweeds) and four control flasks (with only filtered seawater) were used for each pH incubation experiment. The difference between halocarbon concentrations observed between the control and treatment flasks were assumed to be net emissions of halocarbons.

During the experiment, flasks were incubated for four hours (Leedham et al., 2013) in an incubator (Shel Lab) in five different batches at various pH levels; with four replicates for each experiment (unless otherwise stated). We took the quick and simple approach of manipulating the pH via acid/base addition (Schulz et al., 2009). Adjustment of pH to desired values was conducted using a pre-calibrated pH meter (Delta 320, Mettler Toledo) prior to seaweed addition. pH alterations were made using an acid–base titration approach: 1 M sodium hydroxide, NaOH (R & M Chemicals) to increase seawater pH; 1 M HEPES buffer (Sigma-Aldrich) to decrease seawater pH between 8.00 –7.20, at the interval of 0.2 unit. At the end of the four hour incubation period, each flask was gently swirled to get a well mixed sample. The halocarbons were extracted from the seawater sample using a 100 mL glass syringe with metal tip fitted to a Luer tap (Keng et al., 2013). Approximately 40 mL of seawater was collected from each flask.

### Halocarbon analysis

Halocarbons in the seawater were analyzed using a purge-and-trap system and a gas chromatography mass spectrometer (GCMS; Agilent Technologies 7890A GC System; Agilent Technologies 5975C MSD) as described by Hughes et al. (2006). During extraction, the seawater samples were injected into a purging vessel and left to purge for 15 min by oxygen-free nitrogen (OFN) gas at a flow rate of 40 mL min^−1^. This was followed by channelling the gas through a glass wool tube and a molecular sieve to remove excess particles and moisture. The purged gases were then passed on to a Nafion-dryer (Perma-Pure) to a counter-flow of OFN at a rate of 100 mL min^−1^. The halocarbons in the purged gasses were trapped in the coiled section of the stainless steel tubing that was maintained in the headspace of liquid nitrogen at −150 °C by a thermostated heating device. The trapped compounds were subsequently desorbed into the GCMS by swiftly immersing the coiled section into boiling water. A high purity (99.9995%) helium gas (Linde Malaysia) at a flow rate of 1 mL min^−1^ was used as a carrier gas for the analytes into the GCMS system via a transfer line maintained at a temperature of 95 °C. The oven was set to hold for 5 min at 36 °C and then heated to 200 °C with a rate of 20 °C min^−1^, before settling at 240 °C with a 40 °C min^−1^ increase. The detection limits of the system for the halocarbon compounds were all below 10 pmol L^−1^ except for CH_3_I and CH_2_BrI at 30 pmol L^−1^ and 15 pmol L^−1^ respectively, determined through three times the standard deviations of the filtered, autoclaved, pre-purged blank seawater, which was also used for calibration (Abrahamsson & Pedersén , 2000) (view supplementary data, Table S2).

A suite of eight common halocarbons known to be released by tropical seaweeds (Keng et al., 2013) were investigated: CHBr_3_, CH_2_Br_2_, CH_3_I, CH_2_I_2_, CH_2_BrI, CH_2_BrCl, CHBrCl_2_ and CHBr_2_Cl. Two deuterated surrogates (deuterated iodomethane, CD_3_I, and deuterated diiodomethane, CD_2_I_2_) were added as internal standards to monitor and correct for systemic drifts (Leedham et al., 2013). The amount of each halocarbon detected was compared to pre-calibrated standard curves prepared using commercially-available liquid standards diluted in HPLC grade methanol (Fisher Scientific). The identification of each type of halocarbon measured was based on retention time and at least two known mass fragments (Leedham et al., 2013). The fresh weights (FW) of the four seaweed samples were measured before and after the experiments by gentle blotting to remove excess water, while dry weight (DW) was determined 72 h after drying the seaweeds in an oven at 60 °C; until constant mass was reached. These readings were then used to determine the emission rates of halocarbon per seaweed mass.

### Photosynthesis measurements

The state of health of the seaweeds was assessed prior to the experiment using a diving Pulse Amplitude Modulator (PAM) Fluorometer (Zarges 40701 Heinz Walz GMBH) and after the 4 h incubation (Chaloub et al., 2010; Leedham Elvidge et al., 2015; Li et al., 2014). The change in *F*_v_∕*F*_m_ was calculated according to the following formula:

Percentage decrease in *F*_v_∕*F*_m_ (%)

\begin{eqnarray*}= \frac{{F}_{\mathrm{v}}/{F}_{\mathrm{m}}\hspace*{1em}\text{before incubation}-{F}_{\mathrm{v}}/{F}_{\mathrm{m}}\hspace*{1em}\text{after incubation}}{{F}_{\mathrm{v}}/{F}_{\mathrm{m}}\hspace*{1em}\text{before incubation}} \times 100\text{%}. \end{eqnarray*}

The maximum quantum yield (*F*_v_∕*F*_m_) indicates the stress level of the seaweeds prior to and post incubation. This was done by determining the ratio of the difference between the maximum (*F*_m_) and minimum (*F*_v_) fluorescence level to the maximum fluorescence emitted by the seaweed fronds after dark adaptation of seaweeds for at least 15 min using dark leaf clips (Walz, Germany) (Keng et al., 2013). The correlation test was done to determine the effect of seawater pH change on *F*_v_∕*F*_m_ as *F*_v_∕*F*_m_ indicates photosynthetic efficiency of the seaweeds.

### Statistical analysis

Correlation results were obtained using the Pearson Product-Moment correlation analysis on normalized data (Statistica 8.0 software). The significance of seawater pH change on the percentage decrease in maximum quantum yield (*F*_v_∕*F*_m_) of the seaweeds and halogen concentration was determined using one-way ANOVA followed by a post-hoc Tukey test. A significance level of at least *p* < 0.05 was used for all correlation analyses.

## Results

### Halocarbon emissions under various pH levels

In this study, three of the five seaweeds emitted all eight halocarbon compounds with the exception of *K. alvarezii* and *S. binderi,* which did not emit CH_2_I_2_ and CH_3_I, respectively (Table 1; view Table S3 for emissions per fresh weight).

**Table 1:** Selected halocarbon emission rates under varying pH for the five seaweed species studied. All studies were conducted under similar environmental conditions (see ‘Materials and Methods’ section: sample collection and preparation). For each species mean^#^ emission measured in units of pmol g DW^−1^ hr^−1^ ± standard deviation are given, unless emissions fell below the detection limit (see ‘Materials and Methods’ section: Halocarbon analysis); in this case ‘n.d.’ (not detected) is reported. In each case the mean^#^ value is an average of *n* = 4 except *T*. conoides where *n* = 5 biological replicates.

Species	pH	CHBr_3_	CH_2_Br_2_	CH_3_I	CH_2_I_2_	CH_2_BrI	CH_2_BrCl	CHBrCl_2_	CHBr_2_Cl
***Kappaphycus alvarezii***	8.0	9320.2 ± 963.5	139.5 ± 25.4	30.0 ± 3.4	n. d.	34.6 ± 8.8	13.5 ± 2.9	96.7 ± 12.8	439.6 ± 75.2
	7.8	4839.9 ± 980.6	83.7 ± 16.2	35.1 ± 7.2	n. d.	24.4 ± 7.2	11.9 ± 2.2	53.3 ± 13.9	247.2 ± 42.0
	7.6	7689.7± 1836.8	91.1 ± 17.5	26.3 ± 7.5	n. d.	15.6 ± 3.1	13.7 ± 2.1	145.5 ± 31.4	494.0 ± 88.1
	7.4	5987.0 ± 704.7	86.7 ± 17.2	31.6 ± 3.6	n. d.	17.3 ± 3.6	13.3 ± 2.9	117.0 ± 17.1	419.2 ± 48.9
	7.2	6869.0 ± 836.0	107.0 ± 15.5	54.2 ± 9.4	n. d.	19.2 ± 3.5	12.3 ± 1.3	195.8 ± 38.2	585.9 ± 63.5
***Padina australis***	8.0	90.1 ± 36.2	33.7 ± 8.5	3.3 ± 0.8	16.0 ± 5.4	2.6 ± 0.4	7.6 ± 1.6	8.3 ± 7.7	16.3 ± 4.2
	7.8	94.4 ± 80.1	34.9 ± 16.7	2.8 ± 3.7	6.6 ± 2.8	3.3 ± 0.8	9.7 ± 6.8	7.5 ± 11.6	19.8 ± 34.1
	7.6	135.7 ± 48.9	43.6 ± 7.6	1.3 ± 1.7	0.2 ± 0.3	4.4 ± 0.7	16.3 ± 4.1	0.0 ± 0.0	28.7 ± 12.5
	7.4	179.9 ± 81.3	38.5 ± 12.3	3.5 ± 1.3	2.1 ± 2.7	3.5 ± 0.8	6.9 ± 3.3	18.1 ± 7.7	31.9 ± 10.8
	7.2	126.6 ± 33.9	39.4 ± 8.0	46.2 ± 15.0	14.7 ± 12.7	3.9 ± 0.5	16.8 ± 3.9	25.5 ± 6.8	33.6 ± 6.8
***Sargassum binderi***	8.0	400.4 ± 203.8	112.1 ± 72.7	n. d.	8.3 ± 4.7	16.0 ± 10.5	3.1 ± 1.5	14.4 ± 14.4	53.4 ± 35.6
	7.8	214.8 ± 108.4	47.8 ± 8.0	n. d.	3.3 ± 0.4	5.8 ± 1.3	0.8 ± 0.8	9.7 ± 18.2	32.4 ± 20.0
	7.6	162.8 ± 14.7	44.0 ± 4.0	n. d.	5.6 ± 0.8	10.3 ± 1.6	n. d.	6.1 ± 4.9	28.7 ± 4.5
	7.4	653.1 ± 252.0	217.0 ± 131.3	n. d.	10.4 ± 3.9	24.9 ± 12.7	8.0 ± 7.5	27.9 ± 22.4	112.9 ± 63.3
	7.2	281.9 ± 69.6	90.1 ± 55.2	n. d.	8.9 ± 2.9	15.3 ± 4.9	5.8 ± 5.7	12.4 ± 13.0	67.7 ± 23.5
***Sargassum siliquosum***	8.0	3500.4 ± 1079.9	334.6 ± 98.8	11.5 ± 3.6	4.5 ± 4.1	41.2 ± 15.3	100.8 ± 46.1	347.5 ± 172.5	700.4 ± 257.5
	7.8	4856.8 ± 1566.6	355.2 ± 90.8	9.0 ± 2.0	1.2 ± 1.8	33.7 ± 11.2	102.6 ± 25.9	345.2 ± 72.4	832.3 ± 184.4
	7.6	1608.9 ± 249.3	237.1 ± 26.0	18.9 ± 4.4	2.5 ± 0.8	38.4 ± 5.0	114.8 ± 20.2	215.7 ± 32.5	343.7 ± 56.5
	7.4	2808.6 ± 2076.5	404.3 ± 264.4	32.1 ± 18.6	7.9 ± 5.5	50.6 ± 29.8	183.0 ± 127.6	230.5 ± 185.5	499.6 ± 388.1
	7.2	3613.5 ± 1937.3	448.5 ± 257.9	26.6 ± 9.0	6.1 ± 5.4	52.7 ± 23.2	162.7 ± 112.4	273.2 ± 155.5	647.6 ± 344.8
Turbinaria conoides*	8.0	5382.3 ± 1968.9	1857.2 ± 518.3	56.6 ± 11.0	1557.3 ± 610.8	932.7 ± 258.1	156.7 ± 45.8	89.2 ± 47.4	535.7 ± 192.9
	7.8	1664.9 ± 610.4	701.6 ± 502.9	60.8 ± 28.2	671.3 ± 572.3	319.7 ± 255.4	89.7 ± 64.6	24.7 ± 48.2	229.0 ± 103.5
	7.6	3526.8 ± 2326.7	2453.3 ± 600.2	78.3 ± 46.2	1087.3 ± 273.4	909.0 ± 155.6	338.6 ± 87.3	115.6 ± 38.7	629.6 ± 185.6
	7.4	2148.1 ± 170.7	1152.9 ± 180.9	78.0 ± 16.4	606.2 ± 219.7	445.4 ± 88.2	378.4 ± 365.3	39.3 ± 36.9	383.8 ± 59.2
	7.2	5345.6 ± 1936.3	1338.6 ± 391.1	47.9 ± 8.5	230.5 ± 36.3	263.9 ± 48.7	181.8 ± 54.9	110.2 ± 72.1	723.9 ± 267.6

CHBr_3_ was the most abundantly produced compound at all pH levels by all seaweeds, with a maximum production rate of 9,320 ± 964 pmol gDW^−1^ h^−1^ by *K. alvarezii* at pH 8.0. This was followed by CH_2_Br_2_ or CHBr_2_Cl for all species except *T. conoides* that had higher emissions of CH_2_I_2_ instead of CHBr_2_Cl (Table 1).

**Figure 1:**
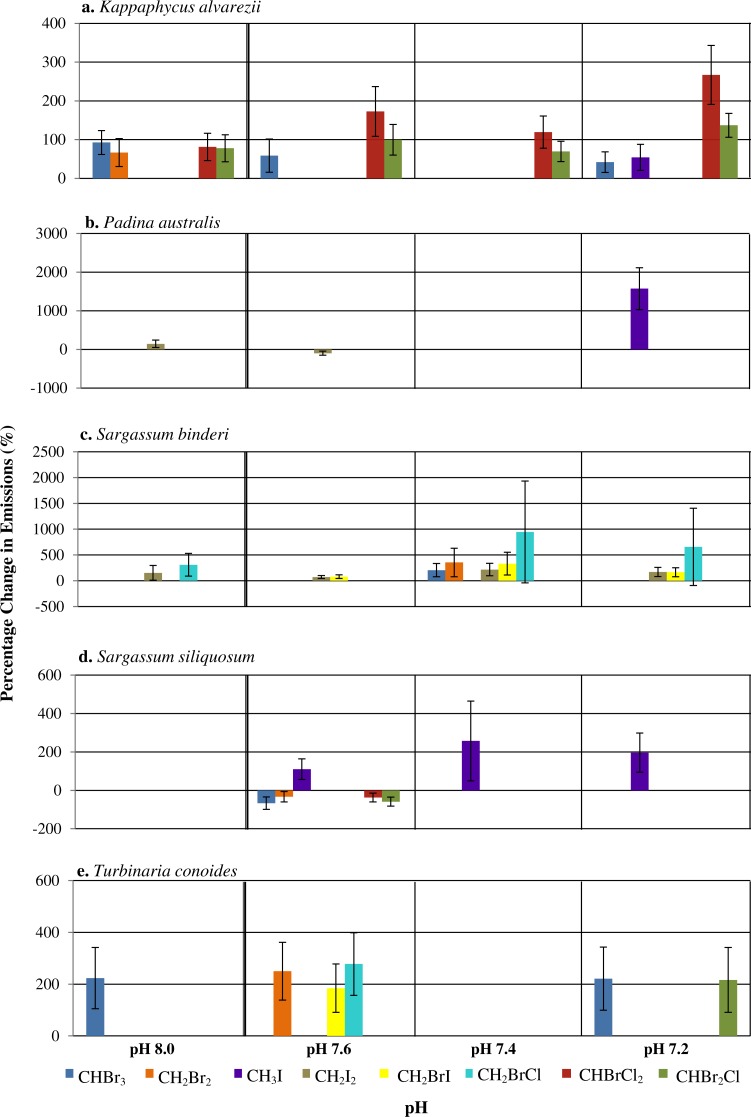
The percentage change (with standard error) of halocarbon emissions by five seaweeds at pH levels relative to ambient pH 7.8. Only significant differences are shown.

A range of responses, depending on seaweed species and individual halocarbons, was observed when seawater pH deviates from the ambient (Fig. 1). Under the various seawater pH treatments, *P. australis* was found to be the least affected seaweed while the rhodophyte *K. alvarezii* was most affected as shown by increases in most of the compounds at all pH levels different from the ambient (Fig. 1). The mixed compounds CHBrCl_2_ and CHBr_2_Cl showed larger significant increase at all altered pH levels, followed closely by CHBr_3_ emission rates that varied at pH 8.0, 7.6 and 7.2. Much higher increases of CHBrCl_2_ and CHBr_2_Cl were observed at pH of 7.4 and 7.2.

Among the phaeophytes, significant increases in the emission of CH_3_I from both *P.  australis* and *S. siliquosum* were observed at pH of 7.4 and 7.2, with a spike in CH_3_I by *P. australis* at the lowest pH, with increased emissions of up to 1,573% (see Table S4). CH_2_BrI emissions increased significantly only at pH levels lower than 7.8 for *S. binderi*. *T. conoides* showed increased emission of CH_2_Br_2_, CH_2_BrI and CH_2_BrCl at pH 7.6. The emission of the dominant biogenic compound (CHBr_3_) at the two extreme pH levels compared to ambient was highest for *T. conoides*.

### Maximum quantum yield (*F*_v_∕*F*_m_)

There were no significant decreases in *F*_v_∕*F*_m_ in relation to decreasing pH for all species, with the exception of *T. conoides* at pH 7.8 (Table S5). In addition, there were no significant correlation between decreasing pH and *F*_v_∕*F*_m_ before and after incubation (Table S6).

### Correlation between pH levels and halocarbon emissions

All halocarbon emission rates fit a normal distribution except for CH_3_I emissions from *P. australis*. Thus, log values of emission rates for this particular data set were used for correlation analysis using the Pearson correlation coefficient (*r*).

#### Correlation between decreasing seawater pH and halocarbon emission rates

CH_3_I, CHBrCl_2_ and CHBr_2_Cl emitted by the rhodophyte, *K. alvarezii* showed negative correlations with decreasing seawater pH values (Table 2). The only positive correlation was for CH_2_Brl (*r* = 0.63; *p* ≤ 0.01) emission. The brominated compounds were not correlated to decreases in seawater pH.

**Table 2:** Pearson Product-Moment Correlation Coefficient analysis between halocarbon emissions produced by five tropical seaweed species at decreasing pH values.

Halocarbon compound	*Kappaphycus alvarezii*	*Padina australis*	*Sargassum binderi*	*Sargassum siliquosum*	*Turbinaria conoides*^d^
CHBr_3_	0.29^NS^	−0.37^NS^	−0.13^NS^	0.15^NS^	−0.03^NS^
CH_2_Br_2_	0.34^NS^	−0.21^NS^	−0.20^NS^	−0.23^NS^	0.11^NS^
CH_3_I	−0.56^a^	−0.62^e^	na	−0.63	0.00^NS^
CH_2_I_2_	na	0.12^NS^	−0.32^NS^	−0.33^NS^	0.66
CH_2_BrI	0.63	−0.47^c^	−0.27^NS^	−0.31^NS^	0.52
CH_2_BrCl	0.06^NS^	−0.39^NS^	−0.27^NS^	−0.37^NS^	−0.25^NS^
CHBrCl_2_	−0.71	−0.57	−0.13^NS^	0.28^NS^	−0.14^NS^
CHBr_2_Cl	−0.53^b^	−0.43^NS^	−0.36^NS^	0.21^NS^	−0.32^NS^

**Notes.**

(*p* ≤ 0.01 unless otherwise stated).

a CH_3_I (*p* ≤ 0.02).

b CHBr_2_Cl (*p* ≤ 0.02).

c CH_2_BrI (*p* ≤ 0.05).

d *n* = 20 but for *T. conoides n* = 25.

e log values for CH_3_I emissions from *P. australis* were used prior to analysis.

NS non-significant.

pH value is changed from 8.0, 7.8, 7.6, 7.4 to 7.2; na, not available

Under decreasing seawater pH, compounds including CH_3_I, CH_2_BrI and CHBrCl_2_ emitted by one or more of the phaeophytes were increased, except for a decreased emission of CH_2_I_2_ and CH_2_BrI by *T. conoides*.

The emission of CH_3_I by the rhodophyte and two of the phaeophytes increased with decreasing seawater pH. However, there was no clear general trend in the emission of CH_2_BrI considering all seaweeds (Table 2).

#### Correlation between *F*_*v*_∕*F*_*m*_ and halocarbon emission rates

There was no significant correlation between percentage decrease in *F*_v_∕*F*_m_ values with halocarbon emissions for all seaweed species, except for CH_2_BrCl emission by *P. australis* (Table S7).

### Effect of pH on halogen composition

The total amount (ng gDW^−1^) of halogens were derived from the sum of bromine, chlorine and iodine (ng) calculated from the respective molar sum. Molar sum of bromine was determined from CHBr_2_, CHBr_3_, CH_2_BrCl, CHBrCl_2_ and CHBr_2_Cl, molar sum of chlorine from CH_2_BrCl, CHBrCl_2_ and CHBr_2_Cl while the molar sum of iodine from CH_3_I, CH_2_I_2_ and CH_2_BrI emitted by the seaweeds (gDW^−1^) into the seawater after the four hour  incubation.

The total amount of halogens emitted by *K. alvarezii, S. binderi* and *T. conoides* showed significant change at pH levels different from ambient seawater pH (Table 3). Both, *K. alvarezii* and *T. conoides*, were observed to emit the highest amounts of halogens at pH 8.0 and 7.6.

**Table 3:** Total amount of halogens emitted per g dry weight of seaweeds (ng gDW^−1^), the amount of bromine (Br), chlorine (Cl) and iodine (I) (ng gDW^−1^) and their percentage contribution (%) to the total halogen amount during the four hour incubation.

pH	Total halogens emitted (ng gDW^−1^)	Br		Cl		I	
		Total amount	%	Total amount	%	Total amount	%
*Kappaphycus alvarezii*							
8.0	9477.5 ± 1132.0^c^	9353.1 ± 1118.0^c^	98.7 ± 0.0^b^	91.7 ± 14.2^a^	1.0 ± 0.1^a^	32.8 ± 4.2^a,b^	0.4 ± 0.1^a^
7.8	4963.0 ± 986.7^a^	4880.9 ± 974.8^a^	98.3 ± 0.2^a,b^	51.9 ± 10.1^c^	1.1 ± 0.2^a,b^	30.2 ± 4.6^a,b^	0.6 ± 0.0^c^
7.6	7937.7 ± 1847.6^b,c^	7803.1 ± 1828.2^b,c^	98.3 ± 0.2^a,b^	113.3 ± 21.4^a,b^	1.5 ± 0.2^b,c^	21.3 ± 3.6^c^	0.3 ± 0.1^a^
7.4	6230.5 ± 732.6^a,b^	6111.1 ± 720.1^a,b^	98.1 ± 0.1^a^	94.5 ± 12.0^a^	1.5 ± 0.1^c^	24.8 ± 1.4^a,c^	0.4 ± 0.0^a,b^
7.2	7279.6 ± 820.5^a,b,c^	7101.9 ± 821.9^a,b,c^	97.5 ± 0.4^c^	140.4 ± 18.8^b^	1.9 ± 0.3^d^	37.2 ± 5.0^b^	0.5 ± 0.1^b,c^
*Padina australis*							
8.0	151.2 ± 53.6^a^	126.6 ± 47.3^a^	83.0 ± 4.1^a,b^	6.4 ± 3.7^a^	4.1 ± 1.2^a,b^	18.2 ± 5.3^a^	12.9 ± 4.4^a,b^
7.8	148.3 ± 121.9^a^	133.1 ± 111.4^a^	86.7 ± 10.0^a,b,c^	6.7 ± 8.2^a^	3.1 ± 2.3^a^	8.4 ± 4.1^a^	10.2 ± 11.2^a,b^
7.6	194.6 ± 67.1^a^	185.9 ± 62.9^a^	95.7 ± 0.6^c^	7.3 ± 2.8^a^	3.7 ± 0.2^a^	1.5 ± 1.4^a^	0.7 ± 0.5^a^
7.4	250.4 ± 107.7^a^	232.6 ± 101.0^a^	92.9 ± 1.5^b,c^	13.2 ± 6.3^a^	5.2 ± 0.5^a,b^	4.6 ± 3.4^a^	1.9 ± 1.3^a^
7.2	229.5 ± 46.5^a^	178.0 ± 38.1^a^	77.5 ± 6.4^a^	14.5 ± 3.0^a^	6.3 ± 0.1^b^	36.9 ± 16.0^b^	16.2 ± 6.4^b^
*Sargassum binderi*							
8.0	528.2 ± 277.0^a,b^	499.8 ± 262.7^a,b^	94.8 ± 1.3^a,b^	11.8 ± 9.0^a^	1.9 ± 1.0^a^	16.6 ± 9.9^a,b^	3.4 ± 1.2^a^
7.8	275.9 ± 135.2^a^	262.2 ± 126.9^a^	95.2 ± 0.9^b^	7.4 ± 7.9^a^	2.2 ± 1.3^a^	6.3 ± 0.9^a^	2.6 ± 0.9^a^
7.6	224.5 ± 20.0^a^	207.8 ± 18.2^a^	92.6 ± 0.5^a^	5.8 ± 2.0^a^	2.5 ± 0.7^a^	10.9 ± 1.7^a,b^	4.9 ± 0.9^a^
7.4	902.6 ± 394.1^b^	855.0 ± 370.7^b^	94.8 ± 0.8^a,b^	24.4 ± 15.6^a^	2.5 ± 0.7^a^	23.2 ± 10.3^b^	2.7 ± 0.8^a^
7.2	410.5 ± 124.4^a,b^	380.4 ± 115.5^a,b^	92.7 ± 1.8^a,b^	13.3 ± 7.2^a^	3.1 ± 0.9^a^	16.8 ± 4.9^a,b^	4.3 ± 1.6^a^
*Sargassum siliquosum*							
8.0	4417.9 ± 1394.6^a^	4174.4 ± 1305.0^a^	94.6 ± 0.9^a^	212.2 ± 89.7^a^	4.7 ± 0.8^a,b^	31.3 ± 13.2^a^	0.7 ± 0.2^b,c^
7.8	5823.3 ± 1681.7^a^	5569.9 ± 1641.0^a^	95.5 ± 0.8^a^	230.5 ± 45.1^a^	4.1 ± 0.7^a^	22.9 ± 8.5^a^	0.4 ± 0.1^b^
7.6	2189.7 ± 289.5^a^	2031.9 ± 278.4^a^	92.8 ± 0.7^b^	126.2 ± 16.4^a^	5.8 ± 0.4^b^	31.6 ± 3.4^a^	1.5 ± 0.3^a^
7.4	3631.4 ± 2665.1^a^	3419.2 ± 2510.7^a^	94.2 ± 0.6^a,b^	162.2 ± 125.5^a^	4.2 ± 0.8^a^	50.0 ± 29.9^a^	1.6 ± 0.4^a^
7.2	4560.5 ± 2448.3^a^	4321.6 ± 2324.3^a^	94.7 ± 0.5^a^	192.4 ± 108.0^a^	4.2 ± 0.5^a^	46.4 ± 17.6^a^	1.1 ± 0.2^a,c^
*Turbinaria conoides*							
8.0	9116.5 ± 2781.1^c^	6947.3 ± 2343.9^c^	75.5 ± 8.3^a^	121.4 ± 43.6^a,b^	1.3 ± 0.4^a^	2047.8 ± 730.3^c^	23.2 ± 8.6^a^
7.8	3201.6 ± 1743.9^a^	2290.5 ± 1030.3^a^	73.6 ± 7.1^a^	51.3 ± 35.8^b^	1.5 ± 0.3^a^	859.8 ± 710.1^a,b^	24.8 ± 7.1^a^
7.6	7434.9 ± 2412.1^b,c^	5689.9 ± 2281.9^a,b,c^	74.9 ± 6.9^a^	167.2 ± 40.6^a^	2.4 ± 1.1^a^	1577.8 ± 339.2^b,c^	22.6 ± 6.4^a^
7.4	4245.0 ± 305.9^a,b^	3261.6 ± 299.0^a,b^	76.9 ± 4.7^a^	117.2 ± 64.9^a,b^	2.7 ± 1.3^a^	866.2 ± 264.2^a,b^	20.4 ± 5.8^a^
7.2	7052.0 ± 2378.5^a,b,c^	6509.4 ± 2284.4^b,c^	92.0 ± 1.6^b^	157.0 ± 64.7^a^	2.2 ± 0.2^a^	385.6 ± 55.9^a^	5.9 ± 1.7^b^

**Notes.**

DW Dry Weight

Values after ± indicate standard deviation between replicates.

*n* = 4 except *T. conoides n* = 5.

a, b, c denote homogenous groups (*p* < 0.05) based on post-hoc Tukey test.

Bromine was the major contributor to the total halogens emitted, with the lowest emission of bromine still above 70%. *Kappaphycus alvarezii* emitted the highest amount of bromine compared to the phaeophytes except at ambient pH, while *T. conoides* emitted significantly higher amount of iodine at all pH levels compared to the other species. The total bromine emitted by *T. conoides* at the two pH extremes was significantly higher than at ambient (Table 3).

Decreasing seawater pH caused increased chlorine emission (*r* =  − 0.89; *p* < 0.01; Table  4) in *K. alvarezii*, while decreasing the emission of total bromine (*r* = 0.85; *p* < 0.01; Table 4). For *T. conoides*, decreasing seawater pH caused a shift from lowered iodine emission to increased bromine and chlorine emission (Table  4).

**Table 4:** Pearson Product-Moment Correlation Coefficient analysis of the percentage (%) contribution of Bromine (Br), Chlorine (Cl) and Iodine (I) in relation to decreasing pH values (8.0–7.2).

	Total mass	% Br	% Cl	% I
*Kappaphycus alvarezii*	0.24^NS^	0.85	−0.89	−0.14^NS^
*Padina australis*	−0.44^NS^	0.08^NS^	−0.60	0.03^NS^
*Sargassum binderi*	−0.18^NS^	0.42^NS^	−0.42^NS^	−0.19^NS^
*Sargassum siliquosum*	0.13^NS^	0.14^NS^	0.14^NS^	−0.53^a^
Turbinaria conoides*	0.15^NS^	−0.59	−0.46^a^	0.62

**Notes.**

NS Non-significant.

*p* < 0.01 unless otherwise stated.

a *n* = 20 but for *T. conoides n* = 25.

## Discussion

### Effect of pH on halocarbon emission

In general, changes of pH lead to changes in halocarbon emissions by tropical seaweeds (Fig. 1). pH changes affected the rhodophyte *K. alvarezii* more than the phaeophytes. *Padina australis* had lowest changes in emissions with changes in pH, except at pH 7.2, when there was a large percentage increase in the emission of CH_3_I. Higher release of a few selected halocarbons, including the iodinated compounds and CHBr_3_ at pH lower than ambient (that is, pH 7.2 and 7.4) by all seaweeds, supports the hypothesis that changes in seawater pH may affect emission of selected halocarbons (Fig. 1). Results also suggest that response to pH changes were species-specific as well as compound-specific. Deviation of pH levels from the ambient 7.8 may affect the chemical bonding between the tertiary structure of the enzymes involved and the H^+^ availability, hence affecting the active site configuration on the enzymes responsible for halocarbon emission in the seaweed species studied (Fersht, 1998). This resulted in higher emission rates of certain halocarbons (e.g., CHBr_3_) by the rhodophyte *K. alvarezii* at pH 8.0 and the phaeophyte *T. conoides* at both pH extremes (Fig. 1). The higher values reported at all pH levels except ambient in this study compared to those from Keng et al. (2013) and Leedham et al. (2013) indicate that the emission of these specific halocarbons is possibly enhanced under lowered pH conditions. There was, however, no uniform trend in the emission of the eight halocarbons by the five seaweeds in response to pH. The ability to tolerate and adapt to environmental changes and oxidative stress varies with species (Abrahamsson et al., 2003; Bischof & Rautenberger, 2012), such as the different strategies adopted by seaweeds from various zonation towards oxidative stress (Dummermuth et al., 2003). Our results concur with these general observations: that the emission under different stresses may be species-specific.

Mtolera et al. (1996) showed that various volatile organic compounds (VOCs) including CHBr_3_, CH_2_Br_2_, CH_2_I_2_, CH_3_I, CHBr_2_Cl, and CHCl_2_Br were released by the red seaweed *Eucheuma denticulatum*, another carrageenophyte which is related to *K.  alvarezii* (Lim et al., 2014), when pH was increased from 8.0 to 8.8 (0.4 interval) and irradiance of 400 µmol photons m^−2^s^−2^ was provided. Similarly, in the present study, increasing the pH to 8.0 resulted in increased emissions of CHBr_3_, CH_2_Br_2_, CHBr_2_Cl, CHBrCl_2_ by *K. alvarezii*. However, the opposite trend (i.e., increased emissions of CH_3_I, CHBrCl_2_ and CHBr_2_Cl) was recorded for this same species when the pH was decreased from 8.0 to  7.2.

The production of the halocarbons is linked to haloperoxidase enzyme activity in seaweeds (Manley, 2002), which is responsible for countering the oxidative stress in seaweeds. Metabolic processes including photosynthesis produce reactive oxygen species, which in high amount, results in oxidative stress (Dummermuth et al., 2003). Compounds like hydrogen peroxide are then involved in the halogenation of organic compounds through enzymatic reactions (Manley, 2002). Haloperoxidases especially the bromoperoxidase, are generally produced by most seaweeds (Butler & Walker, 1993; Carpenter et al., 2000). In the brown seaweeds, haloperoxidases are linked to uptake of seawater iodide in brown seaweeds, transforming it into a strong antioxidant defense system (Küpper et al., 1998). The S-adenosylmethionine (SAM): halide ion methyl transferase reaction is involved in the production of methyl halides, which is not dependent on presence of hydrogen peroxide (Ohsawa et al., 2001; Wuosmaa & Hager, 1990).

Although different enzymes may be responsible for the emission of each halocarbon studied here, the ionization state of acidic and basic amino acids on each of their active sites will be affected by pH. This alteration may modify the various ionic, covalent and disulphide bonds that determine the three-dimensional (3D) structure of the enzymes leading to inactivation due to the inability of substrate molecules to recognize the specific active site of the enzyme (Luk, Loveridge & Allemann, 2015). Tropical seaweed species are reported to contain vanadium based haloperoxidases (Va-HPOs); viz. Va-BPOs present in the red *K. alvarezii* (Kamenarska et al., 2007), the brown Laminariales (La Barre et al., 2010) and the green *Ulvella lens* (Oshiro et al., 1999). Oxidation of the halides Cl^−^, Br^−^ and I^−^ can be catalyzed by chloroperoxidases, halides Br^−^ and I^−^ by bromoperoxidases whereas only I^−^ can be oxidized by iodoperoxidases (Colin et al., 2003; Wever & Van der Horst, 2013). At near neutral pH, vanadate exists in the form of the monoanion (H_2_VO${}_{4}^{-}$) whereas at the extreme of pH 1, the VO${}_{2}^{+}$ is dominant. This positively charged ion is highly stable because of an increased coordination number of the hydrated form as compared to the highly unstable H_3_VO_4_ ion (Crans et al., 2004; Rehder, 2015). Bromoperoxidases can be inactivated in low pH conditions with possible reactivation by vanadate ions (Wever & Kustin, 1990). The existence of various forms of bromoperoxidases is due to acclimatization to various genetic and environmental parameters that influence growth and biochemical processes of seaweeds. The fact that different forms of vanadate gain stability at high, neutral and low pH depending on their respective protonated states shows how changes in seawater pH can greatly influence the mechanism of action for chloro-, bromo-, and iodo-peroxidases that are vanadate dependent. In the present study, the halocarbons CHBr_3_, CH_2_I_2_ and CHBr_2_Cl showed most changes at all pH levels as compared to the ambient (Fig. 1). Further studies are required to confirm the role of bromoperoxidases in these observations.

Since chlorine, bromine and iodine are so prevalent in seawater, the efficient reducing mechanisms for these halogens are essential to prevent an internal accumulation in seaweeds. However, halocarbon emission by the seaweeds is species-specific. Their responses to varying pH levels and photosynthetic performances are also not clearly correlated. For instance the significant negative correlation of CH_3_I with decreases in seawater pH for three of the species studied; *K. alvarezii*, *P. australis* and *S. siliquosum* (ranging from 0.55–0.65; *p* ≤ 0.02) (Table 2) agrees with the fact that this monohalogenated compound could be formed using a specific methyltransferase activity that is more influenced by pH as compared to the Va-BPO reactions. Keng et al. (2013) reported that increase in irradiance resulted in significant changes in the emission rates of CHBr_3_, CH_2_Br_2_, CH_2_BrI and CH_2_I_2_ by *T. conoides*, but not *P. australis*. Only two compounds in *P. australis* showed changes with pH. The fact that emission rates by *P. australis* in both studies (present study and Keng et al., 2013) was least affected by changes in pH and irradiance, respectively, indicates that the haloperoxidase or other enzymatic activities that lead to halocarbon production in certain seaweeds are more stable and unaffected by changes in environmental parameters.

### Relationship between photosynthesis and halocarbon emission under changing pH

As is the case in other photosynthetic organisms, mechanisms to activate quenchers or convert excess absorbed excitation energy into heat are present in seaweeds to prevent the complete reduction of quinone acceptor molecules when an excess of light energy than that required for CO_2_ assimilation has been absorbed (Gorbunov et al., 2001; Lesser & Farrell, 2004). The Mehler reaction which consumes oxygen in the presence of light elevates H_2_O_2_ production whereas the mitochondrial alternative oxidase (AOX) respiration reduces the rate of production (Badger et al., 2000; Czarna & Jarmuszkiewicz, 2005; Lewitus & Kana, 1995; Suggett et al., 2008). Higher irradiance levels correspond to higher amounts of photosynthetic activity which in turn lead to elevated halocarbon emissions (Carpenter et al., 2000; Goodwin, North & Lidstrom, 1997; Keng et al., 2013; Mtolera et al., 1996). However, extremely high light intensities could lead to stress due to intensified production of activated oxygen species, i.e., H_2_O_2_ (Carpenter et al., 2000).

Seawater pH rises due to the photosynthetic activity of seaweeds; this is ensued by a reduction in the partial pressure of CO_2_ (pCO_2_) and bicarbonate ion concentration as CO_2_ assimilation occurs. Hence laboratory incubations in day-time simulations showed a minor increase in pH as seaweed blades absorb CO_2_ for photosynthesis. Conversely respiration takes over when laboratory incubator lights are switched off in night-time simulation, liberating CO_2_ into seawater. Fabricius (2008) reports that this indirectly increases carbonic acid (H_2_CO_3_), bicarbonate ions (HCO${}_{3}^{-}$) and hydrogen ions (H^+^) concentrations while a reduction in carbonate ions (CO${}_{3}^{2-}$) concentration is observed. pH fluctuations as a result of carbonate ions was substantially lessened by the use of HEPES buffer as compared to control samples. In turn, seawater pH variations could impact halocarbon emission levels as the oxidation states of vanadium ions situated at the active sites of Va-BPOs are altered. Incubation studies such as the present one may enable us to understand the linkage between biological processes such as photosynthesis and the mechanisms involved in halocarbon production rates in relation to environmental factors such as pH. Unfortunately, most halocarbon emissions were not affected by photosynthetic efficiency as no significant correlations between percentage decrease in *F*_v_∕*F*_m_ and halocarbon emission rates were recorded; with the exception of CH_2_BrCl emission by *P. australis* (*p* ≤ 0.04; view supplementary data, Table S7).

### Halocarbon production as a defence mechanism

Natural haloperoxidase systems in the brown kelp *Laminaria digitata* produced H_2_O_2_ which can deactivate acylated homoserine lactones that are required for cell-to-cell signalling of certain bacteria; inhibiting biofilm formation hence preventing biofouling on the seaweed exterior (Brochardt et al., 2001). Corpuz, Osi & Santiago (2013) have reported that *S. siliquosum* growing on the seashore of Batangas, Philippines have significant free radical scavenging activity against ROS species namely OH, NO and H_2_O_2_. As concluded by Manley (2002), polyhalomethanes such as the CHBr_2_Cl and CHBrCl_2_ have a particular function mostly as antioxidants but methyl halides such as CH_3_I and CHBr_3_ merely exist as by-products of normal metabolism. Moreover, a strong correlation has been observed between the release of H_2_O_2_ and production of brominated compounds by a phaeophyte and several chlorophytes (Abrahamsson et al., 2003). Hence, there is a link between higher or lower pH, photosynthetic efficiency and H_2_O_2_ production that could also indirectly result in higher halocarbon production rates.

### Effect of pH on halogen composition in emissions from selected seaweeds

Among the seaweeds, *K. alvarezii,* the only commercially cultivated species observed, emitted the largest amount of halogens at all pH levels except for 7.8 (ambient) (Table  3). This was also observed in *T. conoides*, the second largest emitter of all the seaweeds. *T. conoides* is one of the dominant wild species found at the coral reef study site, besides the *Sargassum* species. As these two species are representative of both the wild and farmed species which occur in abundance, the higher contribution of halogen masses from these two species may have certain implications on the environment. Significantly elevated amount of chlorine at all pH levels different from ambient, and the strong negative correlation of the contribution of chlorine with decreasing seawater pH, was also observed for *K. alvarezii* (Tables 3 and 4). Both bromine and chlorine could be implicated in the catalytic destruction of stratospheric ozone. Besides, the halogen atoms in the troposphere could modify alteration of hydroxide and hydroperoxyl radical ratios (OH/HO_2_), or nitrogen dioxide and nitric oxide ratios (NO_2_/NO), which will influence atmospheric oxidation capacity (Stutz et al., 2002; Monks, 2005). The high amount of bromine emitted regardless of pH and the strong negative correlation between decreasing seawater pH and the emission of chlorine by the farmed seaweed, *K. alvarezii,* suggest the need to plan and manage seaweed farming activities through development of adaptation and mitigation strategies in the event of ocean acidification.

At the same time, the high amount of iodine emitted by *T. conoides* could boost formation of ultrafine aerosol particles, affecting the abundance and distribution of cloud condensation nuclei and the atmospheric radiation balance which could lead to changes in local climate (McFiggans et al., 2004; Saiz-Lopez et al., 2012). The role of iodine in the stratospheric ozone depletion has been thought to be negligible due to their short lifespan resulting from rapid loss. However, interest in the role of the short-lived iodine in the upper troposphere/lower stratosphere has increased after studies proved the significance of short-lived bromine contributing to the stratospheric bromine budget (Butz et al., 2009; Dorf et al., 2008).

## Conclusion

Changes in seawater pH resulted in changes in halocarbon emissions by tropical seaweeds. Among the studied species, changes in emissions of halocarbon due to changes in seawater pH was highest for *K. alvarezii* and lowest for *P. australis*. The increased emission of compounds including CH_3_I, CHBrCl_2_, CHBr_2_Cl by *K. alvarezii* with decreasing seawater pH indicates potential acceleration of ocean acidification because of expanding seaweed farming activities in the ASEAN region. Although higher emission rates were observed at the lower pH levels of 7.2 and 7.4 for selected compounds, the emissions were generally species-specific. The change in seawater pH did not significantly affect *F*_v_∕*F*_m_. This shows that *F*_v_∕*F*_m_, and perhaps photosynthesis, may not be closely linked to halocarbon emissions in the five seaweeds studied here.

High amount of bromine was emitted by the seaweeds, particularly *K. alvarezii* (up to 9,353 ± 1,118 ng gDW^−1^; 99% of the total halogen mass). *Kappaphycus alvarezii* also emitted significantly higher amount of chlorine at all pH levels different from the ambient. *Turbinaria conoides* released significantly higher amount of iodine compared to the other seaweeds. Both bromine and chlorine may be contributing to the stratospheric halogen load and play a role in stratospheric ozone depletion. Further studies on emission of iodine from species like *T. conoides* may help to understand the role of inorganic iodine in the upper troposphere/lower stratosphere.

##  Supplemental Information

10.7717/peerj.2918/supp-1 Table S1 Minimum and maximum changes in pH units (pH 7.2 and pH 8.0) taken at hourly intervals for the first 4 h in the 36 h HEPES buffer stability test ^∗^ Seaweed species used is *S. siliquosum*; three replicates used for each treatment method (*n* = 3).

10.7717/peerj.2918/supp-2 Table S2 Halocarbons with their ion fragments, corresponding detection limit and retention timings as detected by the GCMS and the linear regression for their respective calibration curves

10.7717/peerj.2918/supp-3 Table S3 Selected halocarbon emission rates (pmol gFW^−1^ hr^−1^) ±SD under varying pH for the five seaweed species studied All studies were conducted under similar environmental conditions: (A) irradiance of 85 ±5 µmol photons m^−2^s^−1^ photons; (B) Temperature of 30 ±2 °C; (C) average salinity of 30 ±2 PSU; Values before ±indicate average emissions measured in units of pmol gFW^−1^ hr^−1^; Values after ‘±’ indicate standard deviation between replicates; FW, Fresh Weight; SD, standard deviation; n.d., not detected; *n* = 4 except ^∗^ *T. conoides n* = 5.

10.7717/peerj.2918/supp-4 Table S4 Percentage change (%) of halocarbon emissions by five seaweeds at pH levels relative to ambient pH 7.8 Values before ±indicate average emissions measured in units of percentage (%) in comparison to DW emission rates at ambient pH 7.8; negative values represent emissions that are lower than emissions obtained at ambient pH; Values after ‘±’ indicate standard error between respective pH group and pH 7.8 replicates; n.d., not detected; *n* = 4 except *T. conoides n* = 5.

10.7717/peerj.2918/supp-5 Table S5 Percentage decrease (%) in *F*_v_∕*F*_m_ ±S.D. for all seaweed species after 4 hour incubation in seawater of varying pH values. Statistical analysis was conducted using one-way ANOVA and Tukey post-hoc analysis Values before ±indicate percentage decrease in maximum quantum yield (*F*_v_∕*F*_m_) that are obtained by calculating the difference between *F*_v_∕*F*_m_ values prior to and post incubation; followed by division of the *F*_v_∕*F*_m_ prior to incubation and multiplication by 100; Values after the ‘±’ indicate standard deviation between replicates; *n* = 4 except *T. conoides n* = 5; a, b denote homogenous groups (*p* < 0.05) based on post-hoc Tukey test.

10.7717/peerj.2918/supp-6 Table S6 Pearson Product-Moment Correlation Coefficient analysis between changes in seawater pH and percentage decrease in *F*_v_∕*F*_m_ values of five tropical seaweeds *p* ≤ 0.01; *n* = 20; ^∗∗^ for *T. conoides, n* = 25; ^∗^ log values for CH_3_I emissions from *P. australis* were used prior to analysis; NS, non-significant; pH value is changed from 8.0, 7.8, 7.6, 7.4 to 7.2.

10.7717/peerj.2918/supp-7 Table S7 Pearson Product-Moment Correlation Coefficient analysis between halocarbon emissions by five seaweed species and percentage decrease in *F*_v_∕*F*_m_ values after 4 h incubation in different pH levels *p* ≤ 0.01 unless otherwise stated; # (*p* ≤ 0.04); *n* = 20; ** for *T. conoides*, *n* = 25; ^∗^ log values for CH_3_I emissions from *P. australis* were used prior to analysis; NS, non-significant.

## Additional Information and Declarations

### Data Availability

The following information was supplied regarding data availability: The raw data has been supplied as a Supplementary File.
