# Supplementary material for: Halocarbon emissions by selected tropical seaweeds: species-specific and compound-specific responses under changing pH
Source: PeerJ. 2017 Jan 25;5:e2918. doi: 10.7717/peerj.2918 (PMC5270595; doi:10.7717/peerj.2918)
Supplement: Table S1 — ∗ Seaweed species used is S. siliquosum; three replicates used for each treatment method (n = 3). [file peerj-05-2918-s001.docx]

| **Treatment method** | **pH 7.2** | | **pH 8.0** | |
| --- | --- | --- | --- | --- |
|  | **Min** | **Max** | **Min** | **Max** |
| Seawater (no aeration) | -0.04 | -0.08 | -0.02 | -0.05 |
| Seawater (aeration) | -0.02 | +0.06 | -0.02 | -0.06 |
| Seawater + Seaweed (no aeration) | -0.01 | +0.04 | -0.02 | -0.05 |
| Seawater + Seaweed (aeration) | -0.01 | +0.11 | -0.01 | +0.16 |
