# Supplementary material for: Halocarbon emissions by selected tropical seaweeds: species-specific and compound-specific responses under changing pH
Source: PeerJ. 2017 Jan 25;5:e2918. doi: 10.7717/peerj.2918 (PMC5270595; doi:10.7717/peerj.2918)
Supplement: Table S2 [file peerj-05-2918-s002.docx]

| **No.** | **Compound** | **Identifying Ion** | **Retention Time** | **Detection Limit**  **(pmol L^-1^)** | **Linear regression (R^2^)** |
| --- | --- | --- | --- | --- | --- |
| **1.** | CH_2_BrCl | 130 | 10.712 | 7 | 0.986 |
| **2.** | CH_2_Br_2_ | 174 | 12.280 | 1 | 0.996 |
| **3.** | CHBrCl_2_ | 83 | 12.372 | 3 | 0.944 |
| **4.** | CHBr_2_Cl | 129 | 13.959 | 2 | 0.986 |
| **5.** | CHBr_3_ | 173 | 15.392 | 8 | 0.987 |
| **6.** | CH_2_BrI | 222 | 14.204 | 15 | 0.997 |
| **7.** | CH_3_I | 142 | 8.758 | 30 | 0.980 |
| **8.** | CH_2_I_2_ | 268 | 15.909 | 0 | 0.996 |
