# Supplementary material for: Halocarbon emissions by selected tropical seaweeds: species-specific and compound-specific responses under changing pH
Source: PeerJ. 2017 Jan 25;5:e2918. doi: 10.7717/peerj.2918 (PMC5270595; doi:10.7717/peerj.2918)
Supplement: Table S3 — All studies were conducted under similar environmental conditions: (A) irradiance of 85 ±5 µmol photons m−2s−1 photons; (B) Temperature of 30 ±2 °C; (C) average salinity of 30 ±2 PSU; Values before ±indicate average emissions measured in units of pmol gFW−1 hr−1; Values after ‘±’ indicate standard deviation between replicates; FW, Fresh Weight; SD, standard deviation; n.d., not detected; n = 4 except ∗ T. conoides n = 5. [file peerj-05-2918-s003.docx]

| **Species** | **pH** | **CHBr_3_** | **CH_2_Br_2_** | **CH_3_I** | **CH_2_I_2_** | **CH_2_BrI** | **CH_2_BrCl** | **CHBrCl_2_** | **CHBr_2_Cl** |
| --- | --- | --- | --- | --- | --- | --- | --- | --- | --- |
| ***Kappaphycus alvarezii*** | 8.0 | 885.1 ± 206.2 | 13.2 ± 3.7 | 2.8 ± 0.6 | n. d. | 3.3 ± 1.0 | 1.3 ± 0.5 | 9.2 ± 2.4 | 41.8 ± 10.9 |
|  | 7.8 | 459.9 ± 78.6 | 8.0 ± 1.3 | 3.3 ± 0.6 | n. d. | 2.3 ± 0.7 | 1.1 ± 0.2 | 5.1 ± 1.3 | 23.6 ± 3.6 |
|  | 7.6 | 762.7 ± 195.9 | 9.0 ± 1.5 | 2.6 ± 0.8 | n. d. | 1.5 ± 0.3 | 1.4 ± 0.3 | 14.5 ± 3.8 | 49.1 ± 10.6 |
|  | 7.4 | 623.6 ± 86.4 | 9.1 ± 2.2 | 3.3 ± 0.2 | n. d. | 1.8 ± 0.5 | 1.4 ± 0.3 | 12.2 ± 1.7 | 43.6 ± 5.4 |
|  | 7.2 | 701.8 ± 63.1 | 10.9 ± 1.1 | 5.6 ± 1.3 | n. d. | 2.0 ± 0.3 | 1.3 ± 0.1 | 20.3 ± 5.7 | 60.4 ± 10.3 |
| ***Padina australis*** | 8.0 | 16. 5 ± 7.0 | 6.1 ± 1.7 | 0.6 ± 0.1 | 2.9 ± 1.0 | 0.5 ± 0.1 | 1.4 ± 0.3 | 1.5 ± 1.5 | 3.8 ± 1.9 |
|  | 7.8 | 16.8 ± 14.0 | 6.2 ± 2.9 | 0.5 ± 0.7 | 1.2 ± 0.5 | 0.6 ± 0.2 | 1.7 ± 1.2 | 1.3 ± 2.1 | 4.0 ± 5.0 |
|  | 7.6 | 24.8 ± 9.8 | 7. 9 ± 1.6 | 0.2 ± 0.3 | 0.0 ± 0.1 | 0.8 ± 0.2 | 3.0 ± 0.8 | 0. 0 ± 0. 0 | 6.4 ± 3.1 |
|  | 7.4 | 32.8 ± 13.6 | 7.1 ± 2.2 | 0.7 ± 0.3 | 0.4 ± 0.5 | 0.7 ± 0.2 | 1.3 ± 0.6 | 4.2 ± 2.0 | 7.3 ± 3.0 |
|  | 7.2 | 22.5 ± 5.9 | 7.0± 1.3 | 8.3 ± 2.7 | 2.7 ± 2.3 | 0.7 ± 0.1 | 3.0 ± 0.6 | 4.6 ± 1.2 | 7.0 ± 2.2 |
| ***Sargassum binderi*** | 8.0 | 63.9 ± 34.3 | 18.1 ± 12.5 | n. d. | 1.3 ± 0.8 | 2.6 ± 1.8 | 0.5 ± 0.3 | 2.4 ± 2.4 | 8.5 ± 5.9 |
|  | 7.8 | 36.5 ± 22.1 | 7.9 ± 2.2 | n. d. | 0.5 ± 0.1 | 1.0 ± 0.3 | 0.1 ± 0.1 | 1.7 ± 3.2 | 5.5 ± 3.9 |
|  | 7.6 | 26.6 ± 2.9 | 7.3 ± 1.4 | n. d. | 0.9 ± 0.2 | 1.7 ± 0.4 | n. d. | 0.9 ± 0.7 | 4.7 ± 0.5 |
|  | 7.4 | 108.3 ± 38.9 | 35.7 ± 20.8 | n. d. | 1.7 ± 0.6 | 4.1 ± 2.0 | 1.3 ± 1.2 | 4.6 ± 3.6 | 18.6 ± 10.0 |
|  | 7.2 | 47.0 ± 13.0 | 15.2 ± 10.1 | n. d. | 15 ± 0.5 | 2.6 ± 0.9 | 1.0 ± 1.0 | 2.1 ± 2.3 | 11.3 ± 4.1 |
| ***Sargassum siliquosum*** | 8.0 | 582.6 ± 161.3 | 55.9 ± 15.6 | 1.9 ± 0.6 | 0.8 ± 0.7 | 6.9 ± 2.5 | 16.8 ± 7.5 | 57.8 ± 27.8 | 116.4 ± 39.4 |
|  | 7.8 | 818.6 ± 285.0 | 59.5 ± 15.8 | 1.5 ± 0.4 | 0.2 ± 0.3 | 5.7 ± 2.0 | 17.2 ± 4.4 | 57.9 ± 12.8 | 139.9 ± 34.5 |
|  | 7.6 | 263.4 ± 59.5 | 38.4 ± 2.9 | 3.0 ± 0.6 | 0.4 ± 0.1 | 6.2 ± 0.8 | 18.5 ± 2.3 | 35.1 ± 5.6 | 56.3 ± 13.1 |
|  | 7.4 | 460.1 ± 357.6 | 65.7 ± 45.6 | 5.2 ± 3.2 | 1.3 ± 1.0 | 8.2 ± 5.2 | 29.8 ± 21.9 | 37.8 ± 31.7 | 81.9 ± 66.5 |
|  | 7.2 | 552.0 ± 295.7 | 69.0 ± 39.9 | 4.1 ± 1.3 | 0.9 ± 0.8 | 8.1 ± 3.6 | 25.1 ± 17.3 | 41. 9 ± 23.9 | 99.0 ± 52.7 |
| ***Turbinaria conoides**** | 8.0 | 891.9 ± 363.6 | 305.7 ± 93.6 | 9.3 ± 1.6 | 258.2 ± 106.9 | 154.0 ± 47.3 | 25.9 ± 8.5 | 14.7 ± 7.7 | 88.6 ± 35.1 |
|  | 7.8 | 264.3 ± 98.8 | 113.8 ± 87.0 | 9.7 ± 4.7 | 109.1 ± 98.0 | 51.9 ± 44.0 | 14.5 ± 11.2 | 4.2 ± 8.1 | 36.5 ± 17.5 |
|  | 7.6 | 775. 6 ± 610.3 | 517.2 ± 121.4 | 16.0 ± 7.7 | 225.8 ± 39.7 | 190.6 ± 23.9 | 71.2 ± 16.0 | 24.6 ± 8.6 | 136.9 ± 57.4 |
|  | 7.4 | 338.1 ± 42.8 | 181.5 ± 31.1 | 12.3 ± 2.8 | 97.0 ± 42.8 | 70.6 ± 18.4 | 60.2 ± 60.2 | 6.2 ± 5.8 | 60.4 ± 10.9 |
|  | 7.2 | 724.0 ± 236.4 | 181.6 ± 45.6 | 6.5 ± 0.8 | 31.4 ± 4.3 | 35.9 ± 4.9 | 24.7 ± 6.6 | 14.8 ± 9.1 | 97.9 ± 32.2 |
