# Supplementary material for: Halocarbon emissions by selected tropical seaweeds: species-specific and compound-specific responses under changing pH
Source: PeerJ. 2017 Jan 25;5:e2918. doi: 10.7717/peerj.2918 (PMC5270595; doi:10.7717/peerj.2918)
Supplement: Table S4 — Values before ±indicate average emissions measured in units of percentage (%) in comparison to DW emission rates at ambient pH 7.8; negative values represent emissions that are lower than emissions obtained at ambient pH; Values after ‘±’ indicate standard error between respective pH group and pH 7.8 replicates; n.d., not detected; n = 4 except T. conoides n = 5. [file peerj-05-2918-s004.docx]

| **Species** | **pH** | **CHBr_3_** | **CH_2_Br_2_** | **CH_3_I** | **CH_2_I_2_** | **CH_2_BrI** | **CH_2_BrCl** | **CHBrCl_2_** | **CHBr_2_Cl** |
| --- | --- | --- | --- | --- | --- | --- | --- | --- | --- |
| ***Kappaphycus alvarezii*** | 8.0 | 92.57 ± 30.64 | 66.58 ± 36.03 | -14.69 ± 22.59 | n.d. | 41.85 ± 46.74 | 13.25 ± 30.68 | 81.31 ± 35.36 | 77.83 ± 34.82 |
|  | 7.6 | 58.88 ± 43.02 | 8.74 ± 28.51 | -25.00 ± 29.55 | n.d. | -35.96 ± 32.19 | 14.83 ± 25.60 | 172.91 ± 64.30 | 99.82 ± 39.49 |
|  | 7.4 | 23.70 ± 24.95 | 3.50 ± 28.20 | -9.87 ± 22.89 | n.d. | -29.18 ± 33.09 | 11.71 ± 30.50 | 119.47 ± 41.28 | 69.58 ± 26.06 |
|  | 7.2 | 41.92 ± 26.62 | 27.78 ± 26.84 | 54.34 ± 33.58 | n.d. | -21.37 ± 32.88 | 3.28 ± 21.42 | 267.16 ± 76.12 | 137.01 ± 30.79 |
| ***Padina australis*** | 8.0 | -4.48 ± 93.15 | -3.24 ± 53.63 | 19.77 ± 136.47 | 143.78 ± 92.65 | -21.71 ± 26.24 | -21.50 ± 72.12 | 10.88 ± 186.72 | -8.42 ± 131.88 |
|  | 7.6 | 43.83 ± 99.45 | 24.96 ± 52.50 | -54.75 ± 146.65 | -97.52 ± 43.50 | 31.75 ± 31.73 | 67.59 ± 81.85 | 0.00 ± 155.37 | 52.33 ± 142.96 |
|  | 7.4 | 90.63 ± 120.98 | 10.32 ± 59.39 | 27.29 ± 141.46 | -67.71 ± 59.95 | 5.55 ± 34.16 | -29.45 ± 77.88 | 208.47 ± 220.74 | 75.03 ± 148.44 |
|  | 7.2 | 34.10 ± 92.19 | 12.92 ± 52.96 | 1573.20 ± 558.10 | 124.41 ± 198.38 | 16.90 ± 28.26 | 72.39 ± 80.64 | 242.78 ± 179.89 | 71.35 ± 136.16 |
| ***Sargassum binderi*** | 8.0 | 86.39 ± 107.46 | 134.53 ± 153.04 | n.d. | 152.78 ± 142.30 | 176.76 ± 182.55 | 308.09 ± 221.08 | 49.36 ± 240.02 | 64.85 ± 126.03 |
|  | 7.6 | -24.24 ± 50.91 | -7.88 ± 18.63 | n.d. | 71.60 ± 27.96 | 77.45 ± 35.20 | n.d. | -36.83 ± 195.23 | -11.51 ± 63.22 |
|  | 7.4 | 204.02 ± 127.71 | 353.96 ± 275.23 | n.d. | 215.27 ± 119.91 | 329.90 ± 220.62 | 945.69 ± 986.29 | 188.25 ± 298.71 | 248.41 ± 204.79 |
|  | 7.2 | 31.24 ± 59.95 | 88.44 ± 116.68 | n.d. | 170.10 ± 89.46 | 164.88 ± 87.28 | 654.99 ± 750.03 | 27.77 ± 231.68 | 108.90 ± 95.32 |
| ***Sargassum siliquosum*** | 8.0 | -27.93 ± 39.18 | -5.81 ± 37.77 | 27.60 ± 45.95 | 274.07 ± 377.27 | 22.11 ± 56.25 | -1.70 ± 51.53 | 0.66 ± 54.21 | -15.85 ± 38.05 |
|  | 7.6 | -66.87 ± 32.66 | -33.24 ± 26.59 | 110.14 ± 53.38 | 110.44 ± 164.49 | 13.79 ± 36.41 | 11.95 ± 31.99 | -37.52 ± 23.00 | -58.71 ± 23.17 |
|  | 7.4 | -42.17 ± 53.56 | 13.83 ± 78.70 | 257.15 ± 207.95 | 563.03 ± 484.09 | 50.11 ± 94.45 | 78.40 ± 126.93 | -33.24 ± 57.68 | -39.98 ± 51.63 |
|  | 7.2 | -25.60 ± 51.30 | 26.25 ± 76.98 | 196.00 ± 102.08 | 408.18 ± 474.52 | 56.44 ± 76.43 | 58.60 ± 112.41 | -20.87 ± 49.69 | -22.19 ± 46.98 |
| ***Turbinaria conoides**** | 8.0 | 223.29 ± 118.27 | 164.70 ± 73.87 | -6.87 ± 18.15 | 131.98 ± 90.98 | 191.72 ± 80.72 | 74.75 ± 51.02 | 261.63 ± 192.13 | 133.93 ± 84.22 |
|  | 7.6 | 111.84 ± 144.48 | 249.66 ± 111.61 | 28.82 ± 89.00 | 61.97 ± 94.49 | 184.29 ± 93.53 | 277.60 ± 121.10 | 368.54 ± 250.67 | 174.92 ± 92.82 |
|  | 7.4 | 29.02 ± 38.07 | 64.32 ± 76.18 | 28.31 ± 53.68 | -9.69 ± 91.32 | 39.32 ± 84.51 | 321.96 ± 413.66 | 59.30 ± 246.09 | 67.60 ± 52.07 |
|  | 7.2 | 221.08 ± 121.95 | 90.79 ± 90.80 | -21.17 ± 48.45 | -65.66 ± 85.43 | -17.48 ± 81.32 | 102.76 ± 94.57 | 346.80 ± 351.54 | 216.12 ± 125.29 |
