# Supplementary material for: Halocarbon emissions by selected tropical seaweeds: species-specific and compound-specific responses under changing pH
Source: PeerJ. 2017 Jan 25;5:e2918. doi: 10.7717/peerj.2918 (PMC5270595; doi:10.7717/peerj.2918)
Supplement: Table S5 — Values before ±indicate percentage decrease in maximum quantum yield (Fv∕Fm) that are obtained by calculating the difference between Fv∕Fm values prior to and post incubation; followed by division of the Fv∕Fm prior to incubation and multiplication by 100; Values after the ‘±’ indicate standard deviation between replicates; n = 4 except T. conoides n = 5; a, b denote homogenous groups (p < 0.05) based on post-hoc Tukey test. [file peerj-05-2918-s005.docx]

| **pH value** | ***Kappaphycus alvarezii*** | ***Padina australis*** | ***Sargassum binderi*** | ***Sargassum siliquosum*** | ***Turbinaria conoides^*^*** |
| --- | --- | --- | --- | --- | --- |
| **8.0** | 9.1 ± 3.4^a^ | 3.2 ± 1.7^a^ | 11.1 ± 1.1^a^ | 6.4 ± 1.7^a^ | 4.6 ± 1.9^a^ |
| **7.8** | 9.5 ± 5.0^a^ | 3.3 ± 1.0^a^ | 9.7 ± 4.1^a^ | 6.2 ± 2.7^a^ | 13.5 ± 5.9^b^ |
| **7.6** | 9.5 ± 7.5^a^ | 5.0 ± 1.9^a^ | 13.7 ± 7.3^a^ | 9.9 ± 5.9^a^ | 5.7 ± 3.8^a^ |
| **7.4** | 16.6 ± 5.9^a^ | 2.2 ± 1.6^a^ | 13.7 ± 2.4^a^ | 5.6 ± 4.2^a^ | 5.9 ± 2.3^a^ |
| **7.2** | 11.0 ± 3.6^a^ | 3.6 ± 0.9^a^ | 11.8 ± 6.0^a^ | 8.9 ± 4.4^a^ | 4.8 ± 3.6^a^ |
