# Supplementary material for: Halocarbon emissions by selected tropical seaweeds: species-specific and compound-specific responses under changing pH
Source: PeerJ. 2017 Jan 25;5:e2918. doi: 10.7717/peerj.2918 (PMC5270595; doi:10.7717/peerj.2918)
Supplement: Table S6 — p ≤ 0.01; n = 20; ∗∗ for T. conoides, n = 25; ∗ log values for CH3I emissions from P. australis were used prior to analysis; NS, non-significant; pH value is changed from 8.0, 7.8, 7.6, 7.4 to 7.2. [file peerj-05-2918-s006.docx]

| **pH** | ***Kappaphycus alvarezii*** | ***Padina australis**** | ***Sargassum binderi*** | ***Sargassum siliquosum*** | ***Turbinaria conoides***** |
| --- | --- | --- | --- | --- | --- |
| **8.0 - 7.2** | -0.29^NS^ | 0.02 ^NS^ | -0.17^NS^ | -0.16 ^NS^ | 0.21 ^NS^ |
