# Supplementary material for: Halocarbon emissions by selected tropical seaweeds: species-specific and compound-specific responses under changing pH
Source: PeerJ. 2017 Jan 25;5:e2918. doi: 10.7717/peerj.2918 (PMC5270595; doi:10.7717/peerj.2918)
Supplement: Table S7 — p ≤ 0.01 unless otherwise stated; # (p ≤ 0.04); n = 20; ** for T. conoides, n = 25; ∗ log values for CH3I emissions from P. australis were used prior to analysis; NS, non-significant. [file peerj-05-2918-s007.docx]

| **Halocarbon Compound** | ***Kappaphycus alvarezii*** | ***Padina australis*** | ***Sargassum binderi*** | ***Sargassum siliquosum*** | ***Turbinaria conoides***** |
| --- | --- | --- | --- | --- | --- |
| **CHBr_3_** | -0.24 ^NS^ | 0.07^NS^ | 0.04^NS^ | -0.16^NS^ | -0.28 ^NS^ |
| **CH_2_Br_2_** | -0.08 ^NS^ | 0.26^NS^ | 0.12 ^NS^ | -0.04^NS^ | -0.33 ^NS^ |
| **CH_3_I** | -0.07 ^NS^ | 0.07 ^NS*^ | - | 0.14^NS^ | -0.14 ^NS^ |
| **CH_2_I_2_** | - | -0.02^NS^ | 0.20^NS^ | -0.08^NS^ | -0.23 ^NS^ |
| **CH_2_BrI** | -0.09 ^NS^ | 0.39 ^NS^ | 0.19^NS^ | 0.08^NS^ | -0.33 ^NS^ |
| **CH_2_BrCl** | -0.06 ^NS^ | 0.47^#^ | 0.08 ^NS^ | 0.04^NS^ | -0.35 ^NS^ |
| **CHBrCl_2_** | -0.11 ^NS^ | -0.19^NS^ | -0.04^NS^ | -0.06^NS^ | -0.20 ^NS^ |
| **CHBr_2_Cl** | -0.14 ^NS^ | 0.22^NS^ | 0.04^NS^ | -0.10^NS^ | -0.26 ^NS^ |
